# *Drosophila grimshawi* – *Rheb*

**DOI:** 10.17912/micropub.biology.000371

**Published:** 2021-02-16

**Authors:** Chinmay P. Rele, Jared Williams, Laura K Reed, James J Youngblom, Wilson Leung

**Affiliations:** 1 The University of Alabama, Tuscaloosa, AL 35487; 2 California State University Stanislaus, Turlock, CA 95382; 4 Washington University in St. Louis, St. Louis, MO 63130

**Figure 1:**
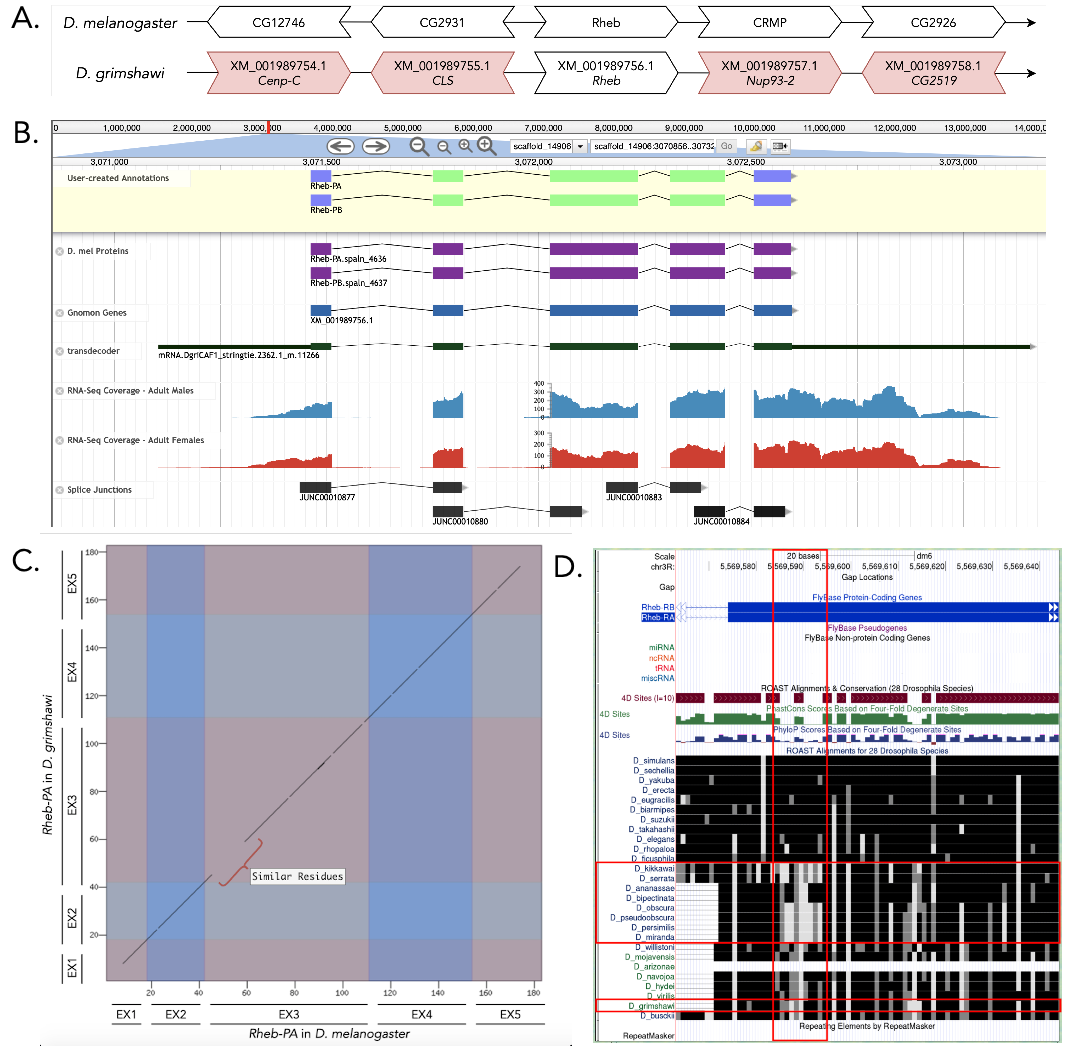
(A) Synteny of genomic neighborhood of *Rheb* in both *D. melanogaster* as well as *D. grimshawi*. (B) Gene Model in Apollo: A screenshot of the Apollo instance housing the gene model, containing student annotations, Dmel proteins, Gnomon annotations, transdecoder genes, RNA-Seq tracks and splice junctions; (C) Dot Plot of gene in *D. melanogaster* (*x*-axis) vs. the gene in *D. grimshawi* (*y*-axis); (D) Low sequence conservation near the start of the third coding exon, which is more obvious in other species, but still existent in *D. grimshawi*.

## Description

Introduction

The Ras homolog enriched in brain (*Rheb*) encodes a Ras homolog (Karassek *et al.* 2010). Ras is family of genes that make proteins involved in cell signaling pathways that control cell growth and cell death (Banerjee and Resat 2016).The gene model reported here (dgri_Rheb) was developed for the May 2011 assembly of *D. grimshawi* Agencourt dgri_caf1/DgriCAF1 (GCA_000005155.1) to describe the ortholog to *D. melanogaster Rheb*(FBgn0041191) at the locus previously annotated as XM_001989756.1. The general protocol and datasets for the genome browser tracks used to establish this reported gene model are reported in Rele *et al.* 2020. Additional tools and resources used in generating and confirming this model include HISAT (Kim *et al.* 2015), BEDTools (Quinlan *et al.* 2010), and the Sequence Read Archive (trace.ncbi.nlm.nih.gov/Traces/sra/?study=SRP073087)*.*

Synteny

The *Rheb* gene on chromosome 3R in *D. melanogaster* is surrounded by the genes *CG12746*, *CG2931*, *CRMP*, and *CG2926*. Upon a *tblastn* search, the *Rheb* ortholog gene in *D. grimshawi*, on scaffold 14906, is surrounded by the genes XM_001989754.1, XM_001989755.1, XM_001989575.1, and XM_001989576.1 (orthologous to *Cenp-C* , *CLS*, *Nup93-2*, and *CG2519)* in *D. melanogaster*, Figure 1A). Though none of these flanking genes appear to be orthologous between the two species, we determined this region to contain the ortholog for *Rheb* in *D. grimshawi* because this location had a substantially better *blastp* hit to the *Rheb* protein sequence than to the second-best hit.

Gene Model

This gene model contains two isoforms of the *Rheb* protein in *D. grimshawi*, Rheb-PA and ­Rheb-PB (Figure 1B). Each of these isoforms contains five identical coding exons. The model in *D. melanogaste*r and *D. grimshawi* have the same length and number of exons, and are similar in peptide sequence. However, there is a dissimilarity at the start of the third exon; but the peptides replacing the ones in *D. melanogaster* have similar properties. The coordinates of the corrected gene model can be found in NCBI at GenBank/BankIt using the accession BK014396 and archived in CaltechData here.

Special Characters of Gene Model

*Improper alignment at start of coding exon three*: As shown in Figure 1D, there is a stretch of amino acids (Figure 1D) that are not identical (black color), but similar (gray/white), between *D. melanogaster* and *D. grimshawi*. This pattern is also evident in a few other species as compared to *D. melanogaster* (Figure 1D). Though this is more obvious in *D. kikkawai, D. serrata, D. obscura, D. pseudoobscura, D. persimilis*, and *D. miranda* (Figure 1D) as compared to *D. grimshawi* (Figure 1D), it nonetheless exists.

*Changes in the splice donor site for coding exon four*: Rheb-PA in *D. grimshawi* has a canonical GT splice donor site at the end of coding exon four. In contrast, the end of coding exon four of Rheb-PA in *D. melanogaster*, and in most of the other *Drosophila* species across the genus *Sophophora*, use a non-canonical GC splice donor site.

Extended data:

Fasta (.faa and .fna) and GFF files are archived and available on CaltechData:D_grimshawi_Rheb_geneModel_gff_faa_fna. This file contains

- Peptide Sequence of *Rheb* in *D. grimshawi*
- Nucleotide Sequence of *Rheb* in *D. grimshawi*
- GFF of *Rheb* in *D. grimshawi*
